# Tuberculin as an Aid to Diagnosis and Treatment

**Published:** 1905-06-03

**Authors:** 


### Hospital Clinics.

### TUBERCULIN AS AN AID TO DIAGNOSIS AND TREATMENT.

Although some early catastrophes threw Koch's tuberculin into bad odour, and resulted for some years in its practical relegation to veterinary diagnosis in this country at least, more extended and better controlled observations leave little doubt that it has a future before it, both in the matter of diagnosis and treatment. Dr. Cranston Low[1] gives an interesting account of its use in the clinic of Professor Neisser at Breslau, where it is employed as a matter of routine for the diagnosis of certain skin diseases. The tuberculin used is the original tuberculin of Koch—that is to say, a broth culture of the organisms filtered free of bacilli and concentrated. The effect of the injection of this substance in a person the subject of tuberculosis is to produce a reaction both local and general. Where the local lesion is superficial as on the skin, the reaction is shown by the swelling and increased warmth of the affected area. The redness extends beyond the actual margin of the visible disease, appears within a few hours of injection, and lasts for 36 or 48 hours. The general reaction is less important from a diagnostic point of view. It consists in an abrupt rise in temperature within six to eight hours of the injection (supposing the subject to be tuberculous), followed by an equally abrupt fall at the expiry of a second similar interval, the body-heat returning to the normal within 24 or 36 hours. The general reaction is not merely a sympathetic phenomenon due to the local changes, for either may occur alone. It is important before using tuberculin to be sure that the lungs are not seriously involved. "If the lung condition is at all marked, it (tuberculin) is best not given at all, as, by producing local reaction in the lung the acute inflammatory process readily leads to a breaking down of the tuberculous tissue and a spread of the disease either locally or into the general circulation. This seems to occur more readily in the lung than elsewhere, as the tissues there are soft and the blood-supply rich." If the lung condition is suspicious it is wise to give the tuberculin cautiously, beginning with one-hundredth of a milligramme, and increasing the dose gradually to one-tenth of a milligramme. If the lungs are healthy the latter dose may be used to begin with. It is recommended that for diagnostic purposes injections should be given every 48 hours until a reaction occurs, the dose rising as follows: One-tenth of a milligramme, one-half, one, five, ten. This last should be the maximum, and if no reaction occurs with it, the result must be accepted as negativing tuberculosis. In lupus vulgaris a reaction usually occurs after the injection of one milligramme.

A good illustration of the value of the substance is offered by the following case. A man, aged 45, presented himself with a large circular diseased area on the right buttock five inches in diameter. It had lasted for 13 years, was bluish-red at the edges, and covered with thick scales and crusts. Over the prominence of the right malar bone was an infiltrated, centrally-depressed, crusted area the size of a sixpenny piece, which had been present for three months. There was no obtainable history of either syphilis or family tuberculosis. Several successive injections of tuberculin resulted in no positive reaction, and a diagnosis of syphilis was made and confirmed by the result of anti-syphilitic treatment.

Dr. Low states that if in any case the local reaction is doubtful, the probability is that the lesion is not tuberculous, for a really positive reaction is unmistakable. Professor Neisser's dictum on the value of tuberculin is as follows:—"Whenever after an injection of tuberculin a typical local reaction occurs, the condition is tuberculous; and whenever, in spite of the administration of suitable doses, it is absent, the condition is non-tuberculous." When used curatively the dosage should be small, for it is important to avoid the production of a profound local reaction. Dr. Low states that lichen scrofulosorum is very amenable to treatment by tuberculin.

[1] Scot. Med. and Surg. Jour., May 1905.

